# Humane Use of Cardiac Puncture for Non-Terminal Phlebotomy of Wild-Caught and Released *Peromyscus* spp.

**DOI:** 10.3390/ani10050826

**Published:** 2020-05-09

**Authors:** Scott C. Williams, Megan A. Linske, Kirby C. Stafford

**Affiliations:** 1 Department of Forestry and Horticulture, Center for Vector Biology and Zoonotic Diseases, The Connecticut Agricultural Experiment Station, PO Box 1106, New Haven, CT 06504, USA; 2 Department of Entomology, Center for Vector Biology and Zoonotic Diseases, The Connecticut Agricultural Experiment Station, PO Box 1106, New Haven, CT 06504, USA; megan.linske@ct.gov (M.A.L.); kirby.stafford@ct.gov (K.C.S.III)

**Keywords:** blood sampling, cardiac puncture, isoflurane, *Peromyscus leucopus*, *Peromyscus maniculatus*

## Abstract

**Simple Summary:**

When researching tick-borne diseases and their management in the interest of improving public health, blood samples often need to be obtained from small rodents, which are the main source of the various pathogens that are picked up by ticks and can infect humans. In such research projects, animals are handled and released back into the environment with the least amount of harm done to ensure their continued survival. Post-sampling animal care is not an option on released animals as it is in a laboratory in a captive setting, therefore, sampling protocols need to reflect this fact. Blood sampling via cardiac puncture (sampling blood directly from the heart) tends to have a negative connotation because it is often associated with a procedure used for humane euthanasia in which sedated animals are bled to death per study protocols. We argue its use for obtaining blood samples is preferred in a field setting in which rodents are released. We show that our recapture and mortality rates rival or are better than other studies that utilize more familiar techniques. Death is not a requirement of its use and we suggest cardiac puncture for blood sampling is in the best interest of animal welfare because it does not make small rodents more prone to infection or negatively impact their vision or survival as can other blood sampling procedures.

**Abstract:**

The cardiac puncture technique for obtaining relatively large volume (50–150 µL) blood samples from sedated rodents has been used in research for nearly a century. Historically, its use to phlebotomize and then release live rodents was more common. However, recently its use in a non-terminal capacity frequently imparts negative connotations in part because exsanguination of sedated animals via cardiac puncture is now an American Veterinary Medical Association-approved euthanasia technique. This association has resulted in ethical concerns by manuscript reviewers and in a few instances, outright refusal by some peer-reviewed journals to publish research that utilized the technique. To counter the perceived negative associations with its non-terminal use, we summarized nearly two decades (2001–2019) of capture and handling data throughout Connecticut, resulting in over 7000 cardiac punctures performed on nearly 5000 sedated, live-captured and released *Peromyscus* spp. We show that our total handling mortality rate (3.7%) was comparable, if not lower, than similar field studies that utilized other phlebotomy techniques. Many public health, integrated tick management, and vector-borne disease ecology studies require samples from individual wild-caught *Peromyscus* spp. over time to determine intervention efficacy and pathogen infection monitoring, and in such field studies, post-operative care is not an option. Proper execution of cardiac puncture does not increase susceptibility of individuals to predation upon release as can potential ocular abnormalities or infections that can occur as the result of use of other techniques. We posit that neither exsanguination nor resulting euthanasia are requirements of cardiac puncture and that its use is entirely appropriate for obtaining blood samples from live-captured and released *Peromyscus* spp. Properly performed cardiac puncture is an excellent technique to obtain blood samples from sedated, individual *Peromyscus* spp. on multiple appropriately-spaced occasions over single trapping seasons while keeping animal welfare a top priority.

## 1. Introduction

Field studies involving zoonotic disease research frequently require tissue sampling from vertebrate reservoirs. Integrated tick management, reservoir-targeted vaccine, zoonotic pathogen management programs, among others, often require ectoparasite sampling, ear tagging, ear tissue biopsies, and relatively high-volume blood samples from small mammals, typically of the genus *Peromyscus* for monitoring or to document treatment efficacy. Institutional Animal Care and Use Committee (IACUC) approvals require that animals endure minimal suffering, stress, and pain in obtaining such samples [1]. Typically, such a sampling regimen would fall under the United States Department of Agriculture Pain and Distress Category D, “animals subjected to potentially painful or stressful procedures for which they receive appropriate anesthetics, analgesics and/or tranquilizer drugs.” While no blood collection method is simple or without minor distress to the animal [2], the proper use of anesthetics can eliminate pain, reduce stress, and permit a variety of phlebotomy techniques to be utilized.

Numerous anesthetic formulations are available for use in sedating small rodents, but working in the field outside of laboratory conditions can somewhat limit their controlled delivery. Ketamine or a combination of ketamine and xylazine hydrochloride can be injected subcutaneously to anesthetize animals for up to 45 min [1,3,4]. This technique requires the use of controlled substances, a needle stick of alert animals, and while it provides a long working window, may result in complications and ethical concerns in the release of partially sedated and defenseless individuals. Inhalant anesthetics such as halothane, methoxyflurane, and isoflurane are more conducive to use in field studies, have rapid induction times, and provide adequate working windows while under sedation, but may have limited availability.

Halothane was introduced in 1956 as a volatile anesthetic for use in major surgeries in humans, but by 1963, at least 350 cases of “halothane hepatitis” caused by putative hepatotoxicity (liver toxicity) were recorded and its use fell out of favor in the United States [5,6]. In a laboratory study on mice, halothane was shown to be a good temporary anesthetic, but was not recommended for extended use because the margin of safety between good surgical anesthesia and death was unacceptably narrow [7]. Methoxyflurane was introduced in 1962 and was proposed as the nontoxic replacement for halothane in humans [6]. It was also very popular in animal research due to its fast induction time, that surgical-level anesthesia was easily maintained, and that it was very difficult, even purposefully, to kill a small mammal with its use [7]. Unfortunately, methoxyflurane also caused unacceptably high rates of liver damage in humans which lead to its demise in the late 1970s, though was still in use in animal research through the early 2000s [6]. As a result, neither halothane nor methoxyflurane are commercially produced in the United States but are still in use in and available from other countries. Isoflurane was introduced in 1979, is inexpensive, and is currently used in veterinary practice and animal research. Induction and recovery times are fast with isoflurane, largely due to its high vapor pressure (239.5 mm Hg at 20 °C) which can result in mortality of research animals due to rapid attainment of fatal concentrations [8]. Methoxyflurane has a more forgiving safety margin as the result of a much lower vapor pressure (22.8 mm Hg at 20 °C) [8] which permits a more gradual induction and lower mortality rates. Since the mid-2000s, the vast majority of field studies in the United States have utilized isoflurane for anesthetizing small rodents.

Because field researchers are, for the most part, limited to isoflurane use only, any tissue sampling techniques that cause pain must be compatible with its use and have to occur quickly during sedation. The most common phlebotomy technique in rodents in the United States is the retro-orbital bleed, executed by puncturing the orbital sinus behind the eye [9]. Using a capillary tube, an experienced investigator can harvest a sufficient volume of blood while inexperience or poor technique can leave the animal blind [9]. In sedated laboratory mice using the retro-orbital bleed, novice personnel were able to successfully obtain an average of 53.9 µL samples, experienced personnel 79.7 µL, but resulted in 43 of 80 mice (54%) with clinical ocular abnormalities thereafter [9]. Fried et al. [9] also reported that this technique has come under increased scrutiny as far as animal welfare due to the perception that it is aesthetically displeasing and because of the high-risk of injury to the eye. While it remains popular in the United States [10,11,12,13,14,15,16], several countries have banned its use because it is considered inhumane [2].

The tail clip is another method recommended where a small piece of the tail is removed and blood harvested from the tail vein [2]. Golde et al. [2] recommended the procedure be done on sedated animals in the event bone tissue is accidentally cut requiring repair. Ethical limitations exist in this technique include the fact that it causes permanent damage to the animal [1]. In order to ensure repeated samplings, a small portion of the tail needs to be excised each time resulting in a small blood volume collected [2], though the tail being shortened by more than 5 mm is not acceptable [17].

Similarly, blood can be sampled indirectly from the lateral tail vein via an incision with a scalpel and capillary tube or directly with a small gauge needle and syringe. Physical restraint of the animal is suggested, but while Golde et al. [2] and Hoff [18] suggested it be done under anesthesia, Parasuraman et al. [1] suggested use of local anesthetic cream and Diehl et al. [17] suggested that anesthesia was unnecessary. Another method is through the saphenous vein, which is found on the lateral aspect of the tarsal joint. It is recommended that the fur be removed to be able to see the vein and blood be withdrawn using a small needle (25–27 gauge) and syringe [17,18].

While these techniques (and others) may be appropriate in laboratory-raised mice in a controlled setting with the ability to check on their welfare and care thereafter, they are not necessarily practical on wild-caught *Peromyscus* spp. while operating in residential driveways in a makeshift mobile laboratory operating out of a pickup truck. While popular, the retro-orbital bleed has the potential to cause harm to the eye which would severely negatively impact survival of individual mice when released back to the environment; over 50% of mice with clinical ocular abnormalities after the procedure is not acceptable in a laboratory setting [9] never mind in the field with no post-operative care. Cutting the tail vein opens up a potential source of infection which we find would not be acceptable in mice released back to the wild. Due to time constraints with field-use of isoflurane, shaving wild-caught *Peromyscus* spp. to expose the saphenous vein is not practical without having to unnecessarily expose individuals to multiple anesthetic doses. In contrast, the cardiac puncture procedure results in a single wound channel with a small gauge needle to a large muscle that closes and clots immediately after withdrawal and poses little risk of infection upon release. It is true that a less invasive wound is required to draw from the tail or the saphenous veins with a needle and syringe, but a high volume of blood can be drawn more quickly directly from the heart [19].

Cardiac puncture-induced exsanguination while under anesthesia is an American Veterinary Medical Association-approved euthanasia technique in small mammals [1,2,17,18,20] and as a result, its modern-day use has seemingly become associated with this procedure only. However, in what appears to be the first description of the cardiac puncture procedure using a needle, tubing, and suction using the mouth, Hicks and Little [21] describe drawing 0.5–1.0 mL blood samples from laboratory-raised house mice (*Mus musculus* L.): “The animals were lightly anaesthetized, fastened on their backs to a small board and swabbed with 70 per cent alcohol prior to the cardiac puncture. Often, after the blood was withdrawn, the mouse would run off the board apparently as well as ever. This procedure was repeated on the same mice, at intervals of two weeks, as many as twelve times and the animals still seemed normal.” Additionally, Diehl et al. [17] mentioned the cardiac puncture procedure “…has been used with recovery in small rodents due to lack of alternative routes.” Similarly, Golde et al. [2] suggested that “The procedure requires a skilled investigator…” some of whom “…are able to keep animals alive for several blood collections.” Additionally, the Canadian Council on Animal Care [22] suggested that the heart is a common blood sampling site in mice.

Curiously, while the majority of the literature suggests cardiac puncture should be used in terminal studies only, both Hoff [18] and Parasuraman et al. [1] state that exsanguination via retro-orbital sinus under sedation can also be used as a terminal technique, yet there is not the same stigma attached to its use. While exsanguination via cardiac puncture under anesthesia is an approved, humane euthanasia technique for rodents when a large-volume blood sample (500+ µL) is required [10,23,24], neither exsanguination nor resulting euthanasia are requirements of this phlebotomy technique.

Cardiac puncture has been successfully used to obtain blood samples from anesthetized white-footed mice (*Peromyscus leucopus* Rafinesque) in Lyme disease-related studies since the late 1980s. Here we focus on our use of cardiac puncture in various field studies throughout Connecticut from 2001–2019 as a non-terminal phlebotomy procedure on wild-caught and released majority *P. leucopus*, which serve as the major reservoir for the pathogens *Borrelia burgdorferi*, *Anaplasma phagocytophilum*, *Babesia microti*, Powassan virus, among others in the Northeast. With this research, we suggest that while some phlebotomy techniques may be appropriate in laboratory settings, they are not necessarily practical in the field where subject animals are released back into the environment and, therefore, cannot receive post-operative care, are more prone to infection, and could be subjected to increased predation rates as a result. We will compare our sampling methodologies, recapture rates, and mortality rates to other field studies to argue that cardiac puncture is the most ethical standard for phlebotomizing sedated, wild-caught and released *Peromyscus* spp. where post-operative care is not an option. Data collected for this work were derived from other previous studies, which is the basis for replacement, refinement, and reduction (the 3Rs) for the ethical use of animals in research.

## 2. Materials and Methods

All methodologies described were approved by the Connecticut Agricultural Experiment Station’s IACUC (S02-17, S03-05, P22-14, P27-16) and the Wildlife Division of the Connecticut Department of Energy and Environmental Protection. From 2007–2019, extensive *P. leucopus* trapping occurred for investigations into the efficacy of various integrated tick management, reservoir-targeted vaccine, landscape management, and reservoir host research projects [25,26,27,28,29,30,31,32]. Trapping occurred in the towns of Guilford, Hampton, Litchfield, Lyme, Mansfield, Norfolk, North Branford, and Redding, CT. Limited *P. leucopus* trapping occurred from 2001–2004 in Cornwall, Groton, Old Lyme, Salisbury, Weston, and Westport with some deer mice captures (*Peromyscus maniculatus* Wagner) in Cornwall and Salisbury. We trapped a total of 42 forested research plots and 162 residences; our research intent was to sample many different locations several times annually rather than the same locations on numerous occasions over the season. Aside from the unavailability of methoxyflurane after the 2004 trapping season, all *Peromyscus* spp. capture and handling protocols were consistent between studies.

Sherman traps (LFAHD folding trap, H. B. Sherman Traps, Inc., Tallahassee, FL, USA) baited with a small amount of peanut butter were set in the afternoon on residential property peripheries or in a grid in wooded areas and collected the following morning, usually late May through early September when overnight lows typically exceeded 15 °C. Traps were retrieved and any non-*Peromyscus* spp. captures were immediately released at the location of capture and documented. All captured *Peromyscus* spp. were returned to a central processing area, either a folding table set up in the field or a portable table mounted to the trailer hitch receiver of a pickup truck when trapping residential properties. If captured *Peromyscus* spp. appeared sluggish and/or stressed due to exposure to precipitation and/or cool overnight temperatures, occupied traps were set in direct sunlight or on the hood or engine block of the parked, non-running vehicle until sufficiently warmed.

In all research projects, any handling technique that caused even minimal pain or stress (e.g., ear tagging, ear tissue biopsy, blood and vibrissae sampling) were always performed when animals were anesthetized. We used the open drop method [18] with methoxyflurane from 2001–2004 and isoflurane from 2007–2019 to temporarily anesthetize *Peromyscus* spp. A cotton ball was soaked with approximately 500 µL of either formulation (and minute amount of mineral oil to reduce isoflurane volatility) and placed in a zippered 30 × 23 cm plastic bag. The bag was then opened and closed several times to allow volatilized anesthetic within to dissipate and then remained closed when unoccupied. The backdoor of occupied traps were inserted into the open plastic bag horizontally, bag was then closed around the trap, the backdoor door was opened fully to depress the trigger to the trap floor to eliminate it as a hiding location, then the trap/bag combination was oriented vertically and given a quick, hard shake as needed until the individual was transferred to the bag. The unoccupied trap was placed on the ground with both doors closed and the bag was sealed and placed on the table. Each *Peromyscus* spp. was then monitored closely and quickly removed from the bag once respiratory rate had slowed to approximately one breath/second. Individual *Peromyscus* spp. were deemed to be at the surgical plane of anesthesia for as long as they remained unresponsive to handling and vibrissae twitching was absent. Anesthetic was applied to new cotton balls and replaced after every four or five sedations or as needed.

Procedures were then executed in descending order of level of pain (first cardiac puncture, then ear biopsy/tag, then vibrissae sample obtained) as surgical anesthesia was maintained. Sedated *Peromyscus* spp. were first placed on the table dorsally recumbent and chest slightly elevated between the thumb and forefinger. With the other hand, the xiphoid process was located and a 1 mL syringe with a 27-gauge, 16 mm needle was slowly inserted below the sternum at a ~20° downward angle ~10–12 mm deep in adults and ~5–8 mm in sub-adults with slight vacuum pressure applied using the plunger. Once blood appeared in the syringe, needle insertion ceased, the appropriate sample volume obtained, and needle then slowly removed. Generally, 50 µL was sampled from juveniles (10–15 g), 100 µL from sub-adults (16–22 g), and 150 µL from adults (23+ g), resulting in <10% total blood volume sampled at any given time based on 72 mL/kg total circulating blood volume in mice (e.g., 1.8 mL total blood volume in a 25 g mouse) [17]. In comparison, Levine [33] suggested that approximately 500 µL of blood could be sampled from *Peromyscus* spp. via cardiac puncture without being fatal. To avoid damaging internal organs and cardiac tamponade (compression of the heart from blood leaking into the pericardial sac) as a result of the procedure, care was taken not to deviate from the established injection channel; once inserted, the needle was never moved laterally, dorsally, or ventrally. While a single needle stick is preferred, in the event a sample could not be obtained, the needle was removed and reinserted as described, but never more than three attempts made. Because we purposefully used a smaller gauge needle than recommended for terminal cardiac punctures, on rare occasions a clot would form within the needle, blocking flow, requiring the use of a new needle and syringe. We followed the cardiac puncture procedure as described in Mills et al. [19] with a smaller gauge (27 as opposed to 22 gauge) and shorter (16 as opposed to 38 mm) needle than recommended.

Immediately after the cardiac puncture, a metal tag (#1005-1, National Band and Tag Co., Newport, KY, USA) was placed deep within the cartilaginous area of the left ear in an attempt to prevent it from being torn out easily. Then if the study required, immediately afterward a 2.0 mm ear tissue biopsy (#13-820-064, Fisherbrand^TM^ Animal Ear Punch, Thermo Fisher Scientific, Inc., Waltham, MA, USA) was taken from the outer portion of the right ear and vibrissae plucked from either side of the face. If an individual broke through the surgical anesthesia plane between procedures, it was placed back into the bag to receive a second dose until surgical anesthesia was again obtained before the necessary procedure was performed. Other procedures that did not cause pain (parasitizing tick sampling, body part measurements) could occur while *Peromyscus* spp. were alert, but it saved time and reduced stress in both animals and researchers to do so while they remained at least partially sedated. Individuals were then placed back into the trap, allowed to fully recover from the effects of anesthesia, and released back to the location from which they were originally captured. In the rare event (<0.1%) an individual remained partially sedated at the time of release, it was covered in a makeshift bed of leaves where it was allowed to recover to lessen the chances of predation on a helpless individual.

Because researchers in the United States are largely restricted to isoflurane use, animals are only at a surgical plane of anesthesia for a short period of time (~1 min) [18]. As a result, it is imperative there be multiple researchers assisting with processes. During the majority of our processing, we had a team of three researchers, one primary handler and two secondary handlers. The primary handler was responsible for sedation, cardiac puncture, ear tagging/biopsy, vibrissae and tick sampling, and obtaining other biological data such as body part lengths, mass, etc. One secondary handler was responsible for pre-loading ear-tagging pliers, ear biopsy pliers (and their sterilization between uses), vertical balance, and ruler which were laid out in order of use while the primary handler was transferring and monitoring sedation of each *Peromyscus* spp. This secondary handler also recorded biological data as called out by the primary handler during processing, as well as capture location, date, etc. The other secondary handler was responsible for transferring blood samples from the syringe to labeled microcentrifuge tubes. In a concerted effort to avoid accidental needle sticks, once blood was obtained by the primary handler, the syringe was laid on the table, the secondary handler picked it up, transferred its contents into a microcentrifuge tube in a rack, put the used, intact syringe in a sharps container, closed the microcentrifuge tube, and placed the blood sample in a rack on ice in a cooler. Ear tissue biopsies, sampled ticks, and vibrissae were also placed in their respective, labeled microcentrifuge tubes by this secondary handler. Such an arrangement with skilled personnel permitted procedures to occur rapidly, methodically, and safely with open communication while keeping animal welfare the top priority.

For our extensive 2007–2019 trapping effort, we quantified total number of trap nights, trapping success, total unique animals processed, number of animals recaptured and reprocessed, and mortality rate to compare our results using cardiac puncture and isoflurane to numerous other published studies similar in scope, but which used other phlebotomy and processing procedures. Lesser data were available for the 2001–2004 effort, but we included results to compare percent mortality using methoxyflurane to isoflurane. All properties were trapped roughly once every two weeks which adhered to guidelines established by Diehl et al. [17] to ensure that no more than 10% blood volume was sampled from the same individual within that time frame. During several capture events in 2019, we timed our procedures for reporting purposes. As soon as individual *P. leucopus* were exposed to isoflurane, a technician started a stopwatch and marked when it achieved the surgical plane, the end of the cardiac puncture procedure, the end of obtaining bodily measurements, and marked the time individuals became alert once again.

## 3. Results

While it varied year to year and increased as we became more proficient, from 2007–2019 we amassed a total of 32,087 trap nights throughout Connecticut. During this effort, we had 7216 successful captures of multiple species (22.5% success), which included 6611 captures (91.6% of total) of 4584 unique *P. leucopus* (Table 1). This resulted in a total recapture rate of 44.2% (2027 recaptures of 4584 unique *P. leucopus*; Table 1); 958 were recaptured once, 351 recaptured twice, 101 thrice, 12 four times, two five times, and one was recaptured on six occasions. We recorded a total of 312 *P. leucopus* mortalities (4.7% of captures) of which 55 were found dead in the trap at time of arrival (0.8%) and 257 died during processing (3.9%) using isoflurane. We also captured 15 different non-target species which were released immediately upon inspection (Table 2).

From 2001–2004, there were a total of 421 *Peromyscus* spp. captures: 383 captures of 320 unique *P. leucopus* and 38 captures of 35 unique *P. maniculatus*. A total of three *P. leucopus* mortalities (0.7% of captures) were recorded: one was found dead in the trap (0.2%) and two died during processing (0.5%) using methoxyflurane. Combined handling mortality of *Peromyscus* spp. anesthetized with both isoflurane and methoxyflurane was 3.7% (259 mortalities from 7032 captures).

Considerable variability existed in the time to achieve surgical plane anesthesia once *P. leucopus* were placed in the bag with isoflurane, but averaged 44.4 s (min 20.8, max 110.0 s). After the surgical plane was achieved, it took an average of 13.5 s (min 11.4, max 16.9) for an experienced researcher to remove the mouse from the bag and obtain a blood sample via cardiac puncture. Ear tagging, ear biopsy, weighing, tick/vibrissae sampling, and body measurements took an average of an additional 30.1 s (min 18.1, max 39.8). Thereafter, individuals remained sedated for an average of 30.2 s (min 0.0, max 110.0) post-processing. This resulted in an average working window of 73.8 s (min 30.7, max 161.2) to accomplish all handling procedures while under sedation with isoflurane.

## 4. Discussion

Cardiac puncture while under anesthesia is a safe and humane method for sampling a relatively large volume (50–150 µL) blood sample from wild caught and released *Peromyscus* spp. Most mentions in the literature suggest that exsanguination via cardiac puncture under sedation can be used for humane euthanasia [1,2,17,18,20] and as a result, its use now seems synonymous with this terminal procedure. Mills et al. [19] expressed reluctance in use of cardiac puncture for blood sampling not because of the terminal procedure association, but because of risk of accidental needle sticks to researchers. They suggested if the procedure must be used, syringes should never be recapped and should be placed in a sharps container immediately after use [19]. Despite its negative connotation, we have shown using cardiac puncture as a phlebotomy technique coupled with a more volatile anesthetic (isoflurane) results in comparable recapture rates and lower handling mortality rates than “preferred” techniques, even when using the more stable methoxyflurane.

In the case of our research protocol, in which mice were sampled and released back to the field, it was not possible to determine post-release mortality of individuals. As a result, we used recapture rate of previously captured individuals to compare to the recapture rate of other published studies using different handling and phlebotomy techniques. Recapture rates are difficult to compare between studies because success is contingent on total number of capture events at single locations as well as available rodent abundances, which can vary considerably year to year based on location, habitat quality, food availability, as well as numerous other environmental factors. However, high recapture success would indicate that previously captured individuals survived handling procedures and were not traumatized enough to avoid being captured again. In an extensive 9 × 9 grid trapping effort, Bunikis et al. [11] used subcutaneous injection of ketamine to sedate *P. leucopus* in Connecticut, obtained blood via retro-orbital sinus bleed, and reported that 62% of animals were recaptured at least once in 1998 and 77% in 1999. In Arizona, Swann et al. [34] captured four different species of *Peromyscus* (*P. boylii* Baird, *P. eremicus* Baird, *P. leucopus*, and *P. maniculatus*) in trapping webs, sedated them with methoxyflurane, and collected ~200 µL blood via the suborbital sinus in treatment *Peromyscus* spp., but not from control *Peromyscus* spp. They reported no difference in recapture rates between treatment (51%) and control (59%) [34]. In an extensive hantavirus surveillance trapping effort in New Mexico using the same trapping web design as Swann et al. [34], Parmenter et al. [35] reported no difference in recapture rates of five *Peromyscus* species (*P. boylii*, *P. eremicus*, *P. leucopus*, *P. maniculatus*, and *P. truei* Shufeldt) sedated with methoxyflurane and bled from the retro-orbital sinus; 48% of individuals were recaptured in control plots and 48% recaptured in treatment locations. In Montana in a 10 × 10 trapping grid, Douglass et al. [13] bled *P. maniculatus* from the retro-orbital sinus without anesthesia and did not bleed others; they recaptured 45% of bled *P. maniculatus* and 46% of non-bled *P. maniculatus*. While neither trapping layout nor bloodletting technique were described, Levin et al. [36] reported a 24% recapture rate for *P. leucopus* in Connecticut. Average reported recapture rate for three grid-trapped California *Peromyscus* spp. (*P. californicus* Gambel, *P. maniculatus*, *P. eremicus*) that were neither anesthetized nor blood sampled was 35% in remnant habitat patches and 43% in highway right-of-ways [37]. Our recapture rate of 44% during our extensive trapping effort (2007–2019) is comparable to these studies and nearly identical to the recapture rate of 43% of Dolan et al. [38], who used a similar residential trapping regimen and cardiac puncture and release with methoxyflurane in Connecticut.

Our direct handling mortality rate (3.9%) using cardiac puncture and isoflurane on 6611 *P. leucopus* captures is comparable, if not better, than other studies that used differing phlebotomy and handling techniques in *Peromyscus* spp. While few published studies report mortality rates, Swann et al. [34] reported 3.9% mortality in methoxyflurane-sedated, suborbital sinus bled *Peromyscus* spp. and 0.7% in control *Peromyscus* spp. that were handled, but neither sedated nor blood-letted. In New Mexico, for *Peromyscus* spp. captured and handled but not blood-letted (control), there was a reported 7.1% mortality rate and for those captured, sedated with methoxyflurane, and blood sampled via retro-orbital sinus, there was a reported mortality rate of 8.6% [35]. Douglass et al. [13] reported 2.7% mortality of *P. maniculatus* that were handled but not sedated or bled and 2.2% in those that were bled via the retro-orbital sinus without anesthesia. Both Swann et al. [34] and Parmenter et al. [35] reported no significant differences in mortality rates in *Peromyscus* spp. that were sedated with methoxyflurane and blood sampled and those that were not. Similarly, Douglass et al. [13] reported no difference in mortality between *P. maniculatus* that were bled without sedation and those that were neither sedated nor bled.

We attributed the overwhelming majority of our mortalities to respiratory arrest from use of isoflurane as most mortalities occurred prior to performing cardiac puncture. Cold or otherwise stressed *P. leucopus* were more often than not unable to be revived from effects of sedation and some *P. leucopus* never stabilized at the surgical anesthesia plane, slipped below, and died. Seldom, if ever that we can recall, did the cardiac puncture procedure itself result in mortality while handling animals. Of the 421 *Peromyscus* spp. captures we preformed cardiac punctures upon while using methoxyflurane, only two mortalities (0.5%) occurred during processing. This is in stark contrast to the 3.9% handling mortality incurred while using cardiac puncture and isoflurane in combination. This is further evidenced by the low reported mortality rates where sedation was not used during handling; 2.2 and 2.7% in Douglass et al. [13] and 0.7% in Swann et al. [34]. The relatively high and anomalous mortality rate of 7.1% of handled and non-sedated *Peromyscus* spp. in New Mexico is coupled with a higher mortality rate of 8.6% in sedated *Peromyscus* spp., suggesting there could have been environmental factors negatively influencing both populations equally [35]. Regardless, the literature shows that there was no difference in recapture or mortality rates of sedated and blood sampled *Peromyscus* spp. and those that were handled without sedation. We report comparable recapture success and comparable, if not lower mortality of *Peromyscus* spp. sedated with methoxyflurane (which is no longer produced domestically) and isoflurane, blood sampled via cardiac puncture, and released.

## 5. Conclusions

Ethical standards and animal welfare should be the utmost priorities in any research programs that utilize animal subjects with the realization that the ability to care for subjects post-treatment is going to differ considerably in laboratory and field studies. In the interest of animal welfare, it is of vital importance researchers and IACUCs alike are able to differentiate between studies such that common, familiar laboratory sampling procedures are not automatically approved for field use, where post-operative care is not an option and animal subjects must rely on their own faculties for survival. In our opinion, the use of cardiac puncture under sedation is the most ethical procedure for obtaining blood samples in wild-caught and released *Peromyscus* spp. The 0.4 mm-wide (27-gauge) needle insertion wound closes and blood clots immediately upon removal resulting in minimal chance of infection and minimal overall negative impact to the well-being of the subject over lancing, tail clipping, or orbital bleeds that have the potential to become infected or negatively impact vision and ultimately, survival. We have shown that cardiac puncture can be used successfully in wild-caught and released *Peromyscus* spp. with similar recapture rates and more importantly, similar, if not lower mortality rates than other past methods used with a more forgiving anesthetic formulation. We feel it is short-sighted for researchers, IACUCs, manuscript reviewers, and journals alike to discount cardiac puncture use altogether largely because its use is associated with an approved euthanasia procedure.

## Figures and Tables

**Table 1:** Total number of unique mice captured, number and percent of those recaptured, total number of captures, total trap nights, and total *Peromyscus leucopus* captures/100 trap nights annually from 2007–2019.

Year	Unique Mice	Recaptures	% Recaptures	Captures	Trap Nights	/100 Trap Nights
2007	147	110	75%	257	900	28.6
2008	199	143	72%	342	1140	30.0
2009	47	14	30%	61	540	11.3
2010	196	130	76%	326	1200	27.2
2011	438	259	59%	697	2256	30.9
2012	196	116	59%	312	1440	21.7
2013	192	121	63%	313	2720	11.5
2014	644	206	32%	850	3816	22.3
2015	442	148	33%	590	3776	15.6
2016	783	217	28%	1000	3022	33.1
2017	734	290	40%	1024	4347	23.6
2018	344	168	49%	512	3906	13.1
2019	222	105	47%	327	3024	10.8
Totals	4584	2027	44%	6611	32,087	20.6

**Table 2:** Number of non-target captures during 2007–2019.

Latin Name	Common Name	# Captures
*Tamias striatus* L.	eastern chipmunk	441
*Blarina brevicauda* Say	northern short-tailed shrew	85
*Microtus pennsylvanicus* Ord	meadow vole	45
*Troglodytes aedon* Vieillot	house wren	9
*Tamiasciurus hudsonicus* Erxleben	American red squirrel	5
*Glaucomys volans* L.	southern flying squirrel	4
*Sciurus carolinensis* Gmelin	eastern grey squirrel	4
*Bufo americanus* Holbrook	American toad	2
*Lithobates sylvaticus* LeConte	wood frog	2
*Mustela erminea* L.	ermine	2
*Thamnophis sirtalis* L.	common garter snake	2
*Didelphis virginiana* Kerr	Virginia opossum	1
*Napaeozapus insignis* Miller	woodland jumping mouse	1
*Ondatra zibethicus* Link	muskrat	1
*Sylvilagus floridanus* J. A. Allen	eastern cottontail	1
